# Design of a Superlubricity System Using Polyimide Film Surface-Modified Poly-Ether-Ether-Ketone

**DOI:** 10.3390/polym17111439

**Published:** 2025-05-22

**Authors:** Yuwei Cheng, Rui Yu, Tingting Wang, Xinlei Gao

**Affiliations:** 1 School of Chemical and Environmental Engineering, Wuhan Polytechnic University, Wuhan 430023, China; yw_123cheng@163.com; 2 Hubei Institute of Aerospace Chemistry Technology, Xiangyang 441003, China; yrsgtc@163.com; 3 School of Materials Science and Engineering, Hubei University, Wuhan 430062, China

**Keywords:** induced alignment superlubricity, liquid crystal, polyimide, poly-ether-ether-ketone, surface modification

## Abstract

Poly-ether-ether-ketone (PEEK) is widely used in dynamic sealing applications due to its excellent properties. However, its tribological performance as a sealing material still has limitations, as its relatively high friction coefficient may lead to increased wear of sealing components, affecting sealing effectiveness and service life. To optimize its lubrication performance, this study employs surface modification techniques to synthesize a thin polyimide (PI) film on the surface of PEEK. When paired with bearing steel, this modification reduces the friction coefficient and enhances the anti-wear performance of sealing components. The tribological properties of a friction pair composed of GCr15 steel and PI-modified PEEK were systematically investigated using a nematic liquid crystal as the lubricant. The friction system was analyzed through various tests. The experimental results show that, under identical conditions, the friction coefficient of the PI-modified PEEK system decreased by 83.3% compared to pure PEEK. Under loads of 5 N and 25 N and rotational speeds ranging from 50 rpm to 400 rpm, the system exhibited induced alignment superlubricity. At 50 rpm, superlubricity was maintained when the load was below 105 N, while at 200 rpm, this occurred when the load was below 125 N. Excessively high rotational speeds (above 300 rpm) might affect system stability. The friction coefficient initially decreased and then increased with increasing load. The friction system demonstrated induced alignment superlubricity under the tested conditions, suggesting the potential application of PI-modified PEEK in friction components.

## 1. Introduction

Tribology, the study of friction, wear, and lubrication, is a key discipline for improving energy efficiency and achieving sustainable development. Although friction is crucial for the proper functioning of mechanical systems, it also results in significant energy losses and material wear. Globally, friction and wear contribute substantially to energy consumption, economic costs, and environmental impact. It is estimated that friction-related issues account for approximately 23% of global energy consumption, imposing an economic burden of around 1.4% of global Gross Domestic Product (GDP) [1,2,3].

To address this issue, the concept of superlubricity has emerged as a novel approach. Superlubricity is a physical phenomenon characterized by an extremely low friction coefficient (μ < 0.01), where friction between two sliding surfaces is nearly eliminated [4]. First proposed by Hirano and Shinjo in 1990 [5], the concept of structural superlubricity was introduced a year later, based on atomic-scale interactions between incommensurate crystal lattices [6]. Subsequent studies have identified superlubricity in various two-dimensional materials, including molybdenum disulfide [7,8,9], boron nitride [10], multi-walled carbon nanotubes [11], and graphite [12,13].

Superlubricity can be broadly categorized into solid superlubricity and liquid superlubricity. In the field of solid superlubricity, Zheng’s research group [14] systematically explored the mechanisms underlying structural superlubricity in materials such as boron nitride [15], graphite [16,17,18], and molybdenum disulfide [19]. Their findings demonstrated the feasibility of achieving superlubricity across different scales, from the nanoscale to the macroscale, by leveraging lattice incommensurability and interface chemical engineering. In contrast, in the domain of liquid superlubricity, Luo’s research group identified various superlubricity systems, including ionic liquid superlubricity [20,21,22], water-based liquid superlubricity [23,24], nanoparticle-enhanced liquid superlubricity [25,26,27,28], liquid crystal superlubricity [29,30], and lubrication systems based on acid–polyhydroxy alcohol mixtures [31,32].

Our research team has also investigated liquid superlubricity and discovered an intriguing phenomenon: induced alignment superlubricity, which occurs in a friction system where a nematic liquid crystal (LC) serves as the lubricant and GCr15 steel and polyimide (PI) act as the friction pair [33,34]. When PI undergoes friction and molecular orientation, the selected LC molecules align with a low pretilt angle nearly parallel to the PI surface while simultaneously orienting along the PI surface grooves. Additionally, the strong interactions between PI and LC molecules facilitate their alignment with corresponding regions on the PI surface. When paired with steel, the electron-rich structure of LC molecules forms coordination bonds with metal ions on the steel surface. In this friction system, the interaction between LC molecules and the surfaces is strong, whereas the intermolecular forces among LC molecules themselves are weak. Consequently, the system’s friction behavior is governed by the movement of oriented LC molecules. The parallel arrangement of LC molecules, coupled with their low viscosity, facilitates induced alignment superlubricity [35].

Liquid crystals (LCs) are intermediate-phase materials that exhibit solid-like properties in directions perpendicular to the surface, preventing direct surface contact and providing high load-bearing capacity. However, in the shear direction, they behave as low-viscosity liquids, effectively reducing the friction coefficient. In this study, we utilize a nematic liquid crystal, a uniaxially ordered phase in which the molecular long axes are predominantly aligned in parallel or nearly parallel orientations. These molecules exhibit weak short-range interactions (van der Waals forces), maintaining high fluidity and low viscosity. The majority of nematic LC molecules possess a rod-like structure, making them easily orientable.

Polyimide (PI), used as the orientation agent, possesses excellent high-temperature resistance, chemical corrosion resistance, orientation stability, electrical insulation, and mechanical strength. Its exceptional thermal stability and mechanical properties allow it to serve as a high-strength orientation film, ensuring the precise and stable alignment of liquid crystal molecules [36,37]. The molecular structure of PI can also be modified to meet specific application needs, making it highly versatile as a structural or functional material. Additionally, different molecular structures result in distinct orientation behaviors.

Poly-ether-ether-ketone (PEEK) is a high-performance thermoplastic polymer widely used in dynamic sealing applications in aerospace, automotive, and petrochemical industries due to its outstanding heat resistance (up to 300 °C), chemical resistance, and mechanical strength. Dynamic sealing components function to prevent the leakage of liquids, gases, or particles between moving interfaces while minimizing friction and wear. They are extensively employed in engines, pumps, valves, and other mechanical systems. Compared to PI, PEEK exhibits superior toughness, dimensional stability, and processability, enabling it to withstand high loads, high-speed motion, and extreme operating conditions. However, PI, as a thermosetting polymer, is inherently brittle and difficult to process, limiting its standalone use in dynamic sealing applications.

Studies have shown that PEEK exhibits relatively high friction coefficients (approximately 0.3–0.4) in dry friction or oil-lubricated environments. Under high loads and high speeds, excessive wear and sealing failure may occur [38,39]. In recent years, researchers have explored various modifications to improve PEEK’s tribological performance, including filler modifications (e.g., MoS_2_, graphite) [40,41,42] and surface coatings (e.g., PVA hydrogels, nanocoatings) [43,44], but significant friction reduction has not been achieved.

Our previous studies have demonstrated that PI exhibits excellent anti-friction properties in specific tribological systems [33,34,35]. Although PI alone is not suitable for dynamic sealing, it can be applied as a surface coating or reinforcement to enhance PEEK’s wear resistance and tribological performance. In this study, we utilize surface modification techniques to synthesize a polyimide (PI) thin film on the surface of PEEK (denoted as PI&PEEK) and design a friction system in which GCr15 steel is paired with PI&PEEK as the friction pair, with a nematic liquid crystal serving as the lubricant. The objective is to enhance the tribological performance of PEEK-based dynamic sealing systems.

## 2. Experimental Section

### 2.1. Experimental Materials

**Scheme 1:**
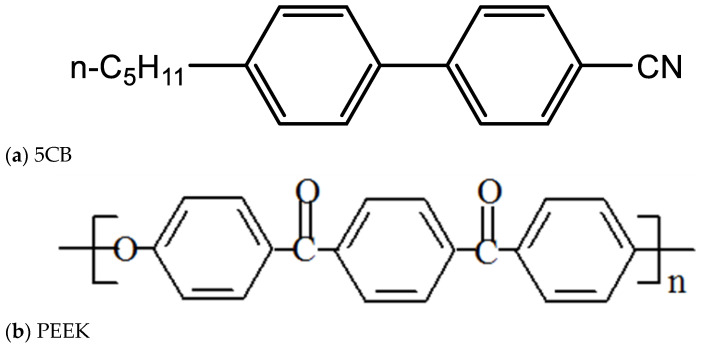

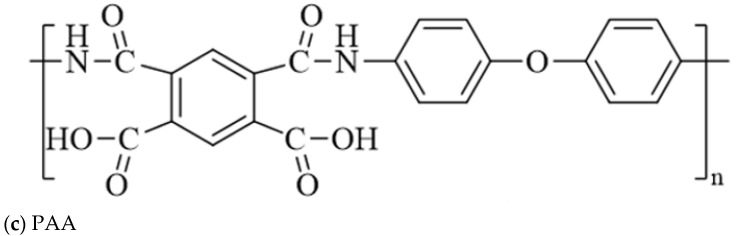
Molecular structures of the experimental materials.

The liquid crystal used as the lubricant in the tests is 4′-pentyl-4-cyanobiphenyl (5CB), purchased from McLin. The GCr15 bearing steel balls (diameter 4.76 mm) were produced by NSK. The PEEK substrate was produced by Guangzhou Tusheng Plastic Products Co., Ltd. (Guangzhou, China) The polyamide acid (PAA) used was sourced from Dalian Jiasheng New Materials Co., Ltd. (Dalian, China). The molecular structures of the materials are shown in Scheme 1.

### 2.2. Preparation of Modified Materials

**Scheme 2:**
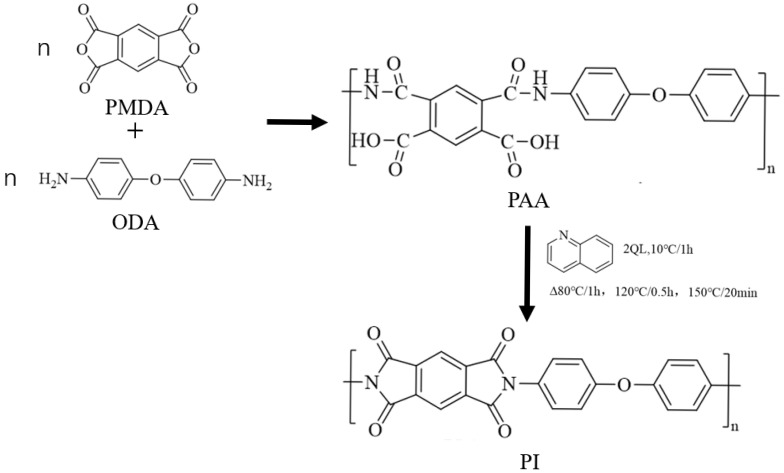
Schematic representation of PI polymerization process.

The purchased PAA was synthesized by the polymerization of diphenylenetetracarboxylic anhydride (PMDA) and 4,4′-diaminodiphenyl ether (ODA). The specific steps for coating PAA onto the PEEK substrate are as follows: Quinoline (QL) was used as a curing catalyst, and QL and PAA were added in a molar ratio of 2 mol of curing catalyst per 1 mol of polyamide acid repeating unit. The mixture was stirred for 1 h to obtain a uniform and stable QL-PAA solution. The surface of the PEEK substrate was polished with 120-mesh sandpaper and cleaned with anhydrous ethanol. The QL-PAA solution was evenly applied to the prepared substrate using a film applicator, with the coating thickness set to 1000 μm. The PAA solution was coated onto the PEEK substrate to form a uniform thin film. The coated sample was dried at 80 °C for 1 h to remove the QL solvent. It was then heated at 120 °C for 0.5 h to initiate the imidization reaction, during which the amide (–CONH–) and carboxyl (–COOH) groups of PAA underwent dehydration condensation to form a five-membered imide ring. The temperature was further increased to 150 °C and maintained for 20 min to accelerate the cyclization reaction, forming a stable polyimide (PI) structure. After cooling, the PI&PEEK sample was obtained. The reaction equation is shown in Scheme 2.

### 2.3. Tribological Testing

#### 2.3.1. Preparation of Friction Pairs

The tribological performance of the systems was tested using a friction and wear tester (UMT-3, CETR, Bruker, Germany) with the rotating module, and 5CB liquid crystal was used as the lubricant. The friction pair selected for the testing was GCr15 bearing steel balls paired with PEEK and PI&PEEK, forming a GCr15/PEEK and GCr15/PI&PEEK face-to-face contact system.

The GCr15 bearing steel balls were used as the static specimen (upper sample). The 1000 µm thick PAA-coated PEEK substrate (PI&PEEK) and the uncoated PEEK substrate were used as the rotating disk samples (lower sample). Before testing, the GCr15 bearing steel balls and the steel tray components of the UMT-3 were cleaned and dried using petroleum ether (60–90 °C) and ultrasonic cleaning. The PEEK and PI&PEEK surfaces were cleaned with anhydrous ethanol and dried. The GCr15 bearing steel balls were fixed on the upper part of the machine, while the PEEK or PI&PEEK samples were mounted on the rotating disk. After assembly, the 5CB liquid crystal lubricant was applied to the rotating disk’s surface, ensuring the lubricant covered the contact area of the friction pair to avoid direct contact between the friction pairs. The testing was performed under face-to-face contact conditions.

Before the friction tests, the GCr15 bearing steel balls were first fixed in the UMT-3, and metallographic sandpaper was affixed to the metal disk’s plane with double-sided tape. The metal disk was fixed on the UMT-3, and the GCr15 steel ball was polished to a smooth surface under dry conditions using the UMT-3. The specific procedure was as follows: first, using a 5 N load and 60 rpm speed on 180-mesh sandpaper for 5 min; then, rotating the upper specimen 90° and replacing the sandpaper with a new 180-mesh sandpaper, continuing under the same conditions for another 5 min. Next, the upper specimen was rotated 180°, and 1000-mesh sandpaper was applied, followed by polishing for 3 min. Lastly, the specimen was rotated 90° again, replacing the sandpaper with 2000-mesh and continuing to polish for 5 min under the same testing conditions.

#### 2.3.2. Friction Testing

##### Selection of Different Plates and Lubricants for Testing

The single-molecule liquid crystal 5CB was used as the lubricant for the tribological tests. The test temperature was set to 25 °C, and a 5 N load was applied with a rotational speed of 200 rpm (0.18 m/s). The testing time was 3600 s.

##### Different Speed Tests

The single-molecule liquid crystal 5CB was used as the lubricant for the tribological tests. The test temperature was set to 25 °C, and a 5 N load was applied with a rotational speed of 200 rpm (0.18 m/s). The testing time was 3600 s.

##### Different Load Tests

The single-molecule liquid crystal 5CB was used as the lubricant for the tribological tests. The test temperature was set to 25 °C, and a 5 N load was applied with a rotational speed of 200 rpm (0.18 m/s). The testing time was 3600 s.

### 2.4. Peel Strength Test Method

The peel strength between PI and PEEK was tested using a high-low temperature material testing machine (68TM-10, Instron, Norwood, MA, USA) at 25 °C. The test was conducted according to the ASTM-D3330-22 standard [45], which is used to test the peel strength of pressure-sensitive adhesive tapes. This standard is mainly used to evaluate the adhesion of tapes to different substrates by measuring the force required to peel the tape from a specific surface. According to Method A of the standard, which is suitable for testing the adhesion of tapes to rigid substrates, a 180° peel test was conducted at a peel rate of 300 mm/min to ensure the standardization and comparability of the test data.

### 2.5. Surface Analysis Methods

The surface of the PI (PMDA-ODA)/PEEK film samples before friction, after dry friction under a 5 N load, and after liquid crystal lubrication testing was analyzed using an AE-100M white light interferometer (Taizhi Precision Technology Co., Ltd., Suzhou, China). The liquid crystal lubrication test conditions were the same as in Load of 25 N Section. The Raman spectra of the PI film surface before and after liquid crystal lubrication testing were detected using a DXR Raman microscope (Thermo Fisher Inc., Waltham, MA, USA), with the test conditions the same as in Load of 25 N Section.

## 3. Results and Discussion

### 3.1. Micro-Friction Experiments

**Figure 1:**
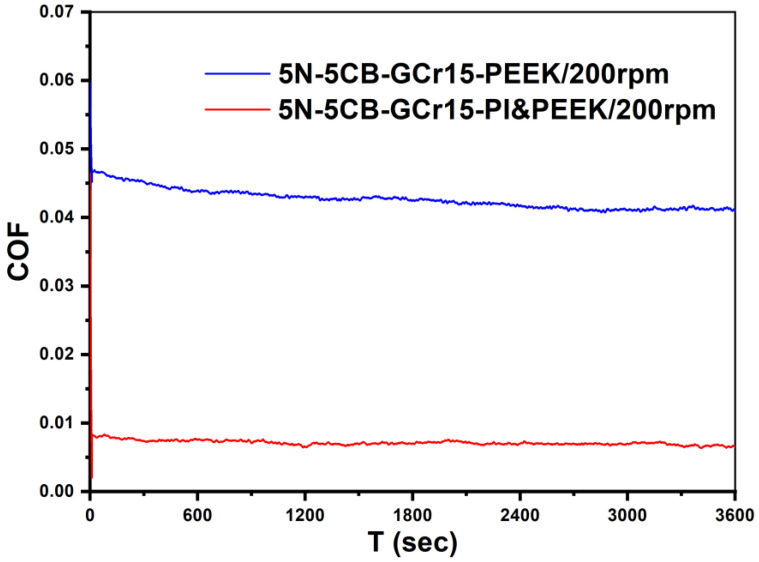
Friction coefficients as a function of time using 5CB as the lubricant and GCr15/PEKK or GCr15/PT&PEKK as the friction pair.

The reason for choosing 5CB as the lubricant and PI (PMDA-ODA) as the friction component in this study is that the pretilt angle between 5CB and PI (PMDA-ODA) is very small (approximately 1.0°) [34], and 5CB molecules are almost parallel to PI (PMDA-ODA) molecules. As shown in Figure 1, when 5CB was used as the lubricant, the coefficient of friction (COF) of the friction system using PI&PEEK as the lower sample was 0.007158, while the COF of the system using PEEK as the lower sample was 0.04268 under the same test conditions. The COF of the friction system using PI&PEEK was only 1/6 of that using PEEK, indicating that the COF of the friction system decreased by 83.3% after the addition of the PI film. This demonstrates that PI plays an important role in the lubrication of the friction system. PI has an excellent ability to regulate liquid crystal orientation. During friction, specific microstructures or grooves are easily formed on the PI surface, and the anchoring energy between the PI surface and the 5CB liquid crystal molecules promotes the orientation of the liquid crystal molecules along the friction direction. Since the pretilt angle between 5CB and the PI used is small (approximately 1.0°), the 5CB molecules are almost parallel to the PI surface. When the 5CB molecules are aligned in a specific direction, a stable lubricating film is formed, reducing friction and lowering the COF. Additionally, the weak intermolecular forces between the 5CB molecules and their parallel alignment maintain the high fluidity and low viscosity of the liquid crystal, further reducing the COF. Therefore, the orientation effect of PI on the liquid crystal effectively promotes superlubricity, and the COF of the system is significantly lower than that of the system using PEEK alone. In summary, the GCr15/PI&PEEK friction system can achieve superlubricity when 5CB is used as the lubricant, with PI playing a key role in lubrication and PEEK providing support with its excellent toughness and impact resistance, maintaining the stability of the friction system.

#### 3.1.1. Speed Variation Tests

The effect of speed on the friction performance of the system was investigated under fixed loads of 5 N and 25 N. The speed was increased from 50 rpm in increments of 50 rpm, with each test cycle starting and stopping, until the speed reached 400 rpm. The COF of the GCr15/PI&PEEK friction system lubricated with 5CB was tested.

##### Load of 5 N

**Figure 2:**
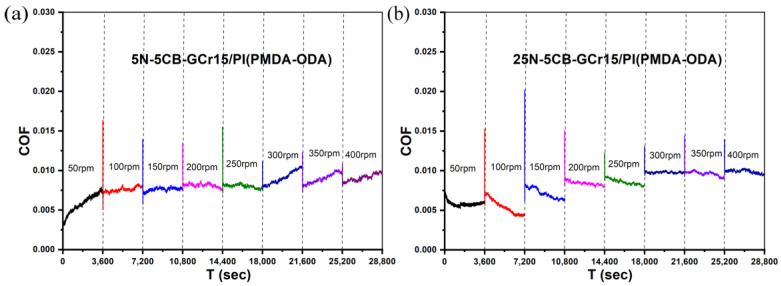
Friction coefficients as a function of time using 5CB as the lubricant and GCr15/PT&PEKK as the friction pair with different speeds. (**a**) COF curves under a 5 N load; (**b**) COF curves under a 25 N load.

As shown in Figure 2a, in the GCr15/PI&PEEK friction system lubricated with 5CB, when the load was kept constant at 5 N, the COF of the system gradually increased as the speed increased from 50 rpm to 400 rpm, but the system remained in a superlubricity state. When the speed was between 100 rpm and 250 rpm, the COF remained stable between 0.007 and 0.008. However, when the speed was below 50 rpm or above 300 rpm, the COF curves in each test cycle showed significant fluctuations and a monotonic upward trend, indicating that excessively low or high speeds can affect the stability of the friction system, leading to an increase in the COF. Nevertheless, the system remained in a superlubricity state.

##### Load of 25 N

As shown in Figure 2b, under the same conditions as the previous experiment but with the load increased to 25 N, the COF of the system also showed a gradual increase with increasing speed, but the system remained in a superlubricity state. Compared to the experiment with a 5 N load, the COF of the system under a 25 N load was generally higher, and the COF curves in each test cycle were more stable. This indicates that increasing the load may lead to an increase in the COF, but at higher loads, the effect of speed on the stability of the friction system is less pronounced.

#### 3.1.2. Load Variation Tests

The effect of load on the friction performance of the system was investigated using 5CB as the lubricant. The tests were conducted at fixed speeds of 50 rpm and 200 rpm, with the load increased from 5 N in increments of 10 N. The COF of the GCr15/PI&PEEK friction system lubricated with 5CB was tested under different loads. Since excessive loads can cause the PI film to rupture, the load was increased gradually, with the maximum load being the limit that the PI film could withstand.

##### Fixed Speed of 50 rpm

**Figure 3:**
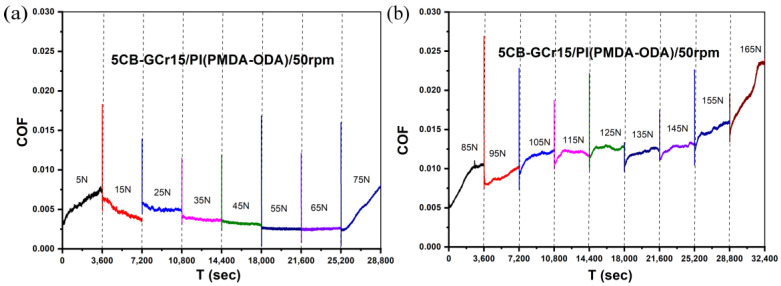
COF as a function of time using 5CB as the lubricant and GCr15/PT&PEKK as the friction pair with different loads at 50 rpm. (**a**) COF from 5 N to 75 N; (**b**) COF from 85 N to 165 N.

As shown in Figure 3, at a fixed speed of 50 rpm, as the load increased from 5 N to 165 N, the COF of the friction system first decreased and then increased. The process can be divided into three stages: the liquid crystal lubrication orientation stage (5–25 N), the liquid crystal lubrication stabilization stage (35–65 N), and the boundary lubrication stage (75–165 N). The friction system remained in a superlubricity state when the load was between 5 N and 95 N. When the load exceeded 105 N, the system lost its superlubricity state, and when the load reached 175 N, the PI film ruptured. Therefore, the maximum load-bearing capacity of the system was 165 N (bearing pressure of approximately 36.4 MPa). As the load increased from 5 N to 65 N, the COF of the system decreased from 0.005837 to 0.002525, and the system remained in a superlubricity state. When the load increased from 75 N to 165 N, the COF increased from 0.004916 to 0.019361. The system remained in a superlubricity state during the entire test cycle at 75 N and during the first half of the test cycles at 85 N and 95 N. When the load exceeded 105 N, the system lost its superlubricity state. Friction caused the PI molecular structure to orient, and the reoriented PI molecules combined with the liquid crystal molecules to form a lubricating film, reducing the COF. However, the COF curves at 5 N, 15 N, and 25 N were unstable, indicating that the lubricating film was unstable during the friction process. This stage is referred to as the orientation stage. From 35 N to 65 N, the COF gradually decreased, and the lubrication system was very stable, indicating that the lubricating film formed quickly and remained stable in this load range. This stage is referred to as the stabilization stage. However, when the load increased from 75 N to 85 N, the COF gradually increased and showed significant fluctuations, indicating that the lubrication mode transitioned from liquid lubrication to boundary lubrication. At high loads, the lubrication area of the lubricating film was gradually compressed, and the contact area between the steel ball surface and the PI film increased. From 95 N to 145 N, the system was in a stable boundary lubrication state, and the lubrication area of the lubricating film remained stable, with little change in the COF. From 155 N to 165 N, the lubrication area of the lubricating film further decreased, and at 175 N, the lubricating film ruptured, causing excessive friction that tore the PI film. This stage is referred to as the boundary lubrication stage. In summary, when the load was below 65 N, the system was in a liquid crystal lubrication state, and the lubricating film formed by PI-induced liquid crystal orientation effectively lubricated the friction system. The stage from 5 N to 25 N is the orientation stage, and the stage from 35 N to 65 N is the stabilization stage. When the load exceeded 65 N, the system gradually entered the boundary lubrication stage, and the lubrication effect of the lubricating film gradually decreased. Although the system was no longer in a superlubricity state, the COF remained relatively low. At the maximum load of 165 N, the COF was 0.01936.

##### Fixed Speed of 200 rpm

**Figure 4:**
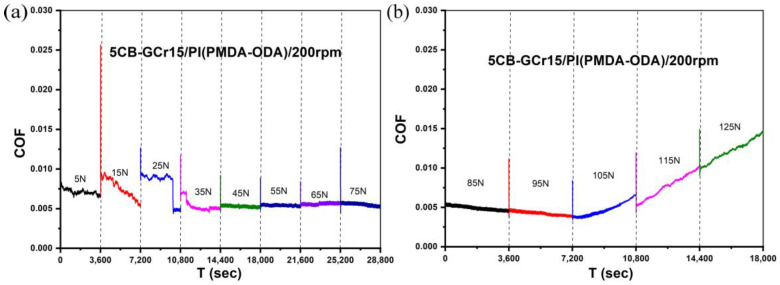
COF as a function of time using 5CB as the lubricant and GCr15/PT&PEKK as the friction pair with different loads at 200 rpm. (**a**) COF from 5 N to 75 N; (**b**) COF from 85 N to 125 N.

As shown in Figure 4, at a fixed speed of 200 rpm, the COF curves of the system showed a trend of first decreasing and then increasing. The process can also be divided into three stages: the liquid crystal lubrication orientation stage (5–25 N), the liquid crystal lubrication stabilization stage (35–95 N), and the boundary lubrication stage (105–125 N). The friction system remained in a superlubricity state when the load was between 5 N and 115 N. When the load reached 125 N, the system lost its superlubricity state (COF = 0.01218), and at 135 N, the PI film ruptured. Therefore, the maximum load for the test was 125 N (bearing pressure of approximately 27.6 MPa). As the load increased from 5 N to 25 N, the COF of the system gradually increased from 0.007158 to 0.008187, and the friction process showed increased fluctuations. As the load increased from 35 N to 95 N, the COF decreased from 0.005349 to 0.004245, and the friction process was smooth. As the load increased from 105 N to 125 N, the COF increased from 0.004733 to 0.012181. Compared to the results of the experiment inFixed Speed of 50 rpm Section, the liquid crystal lubrication orientation stage was the same (5–25 N). However, the liquid crystal lubrication stabilization stage in this experiment was longer, with the maximum load reaching 95 N, indicating that the liquid crystal lubricating film is more easily maintained at higher speeds. The boundary lubrication stage in this experiment was shorter, with the maximum load being only 125 N. This is because, after the system transitioned to boundary lubrication, part of the steel surface came into direct contact with the PI film, causing wear. At higher speeds, the friction distance increased, leading to more wear on the PI film.

### 3.2. Peel Strength Test of PI&PEEK

**Figure 5:**
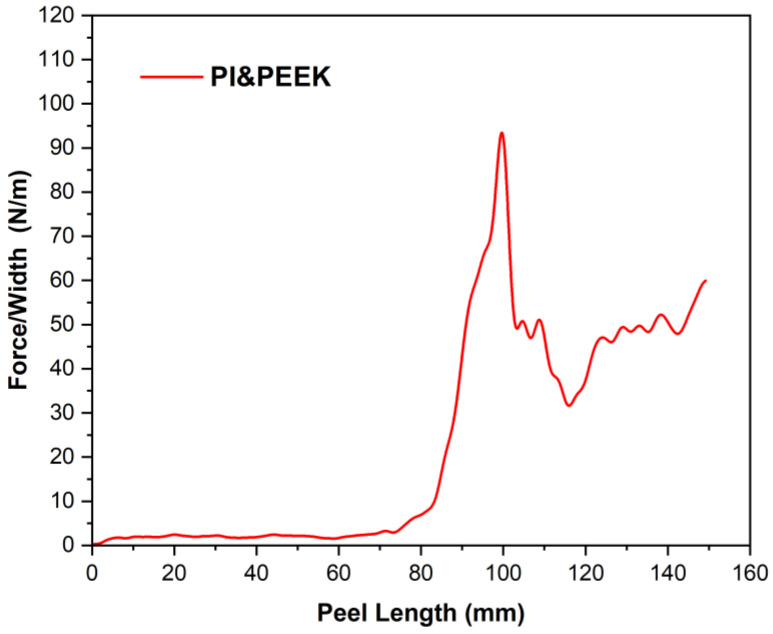
The peel force versus peel length curves for PI&PEEK composites.

The test results are shown in Figure 5. At room temperature, the maximum peel force/width between PI and PEEK was 93.348 N/m, indicating a high interfacial adhesion between the PI film and the PEEK substrate. However, for seals operating under high temperatures (>250 °C), high loads (>30 MPa), and long-term reciprocating motion, the peel strength requirements should be higher. These results show that the PI&PEEK composite material prepared by our modification method has good interfacial stability and potential as a sealing material. However, to ensure long-term stability and durability in typical operating conditions (e.g., hydraulic system seals, cylinder seals), the interfacial adhesion between PI and PEEK can be further improved by optimizing the process (e.g., plasma treatment, interfacial enhancers, nanofillers). Since the focus of this experiment was on the study of superlubricity in the friction system, we did not extensively investigate the interfacial adhesion between PI and PEEK. In future experiments, we will use plasma activation to increase the polarity of PEEK, making it easier to form strong interfacial bonds with PI and further improve interfacial adhesion.

### 3.3. Characterization of PI Film Samples

#### 3.3.1. White Light Interferometry

**Figure 6:**
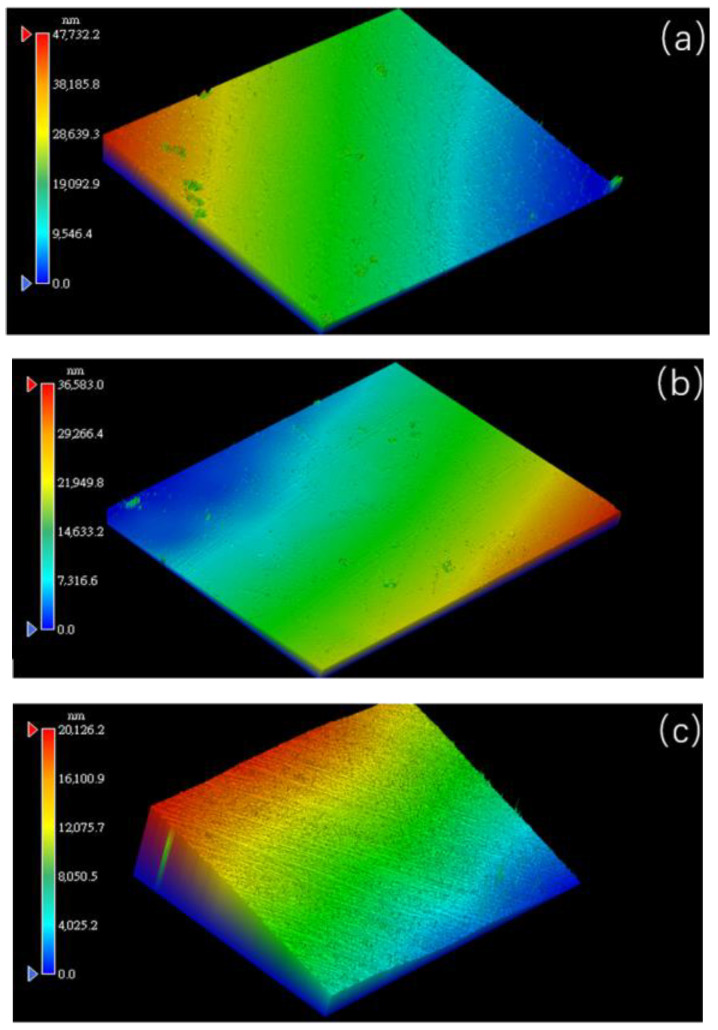
White light interferometry images of the PI film. (**a**) Original PI; (**b**) wear scar after dry friction; (**c**) wear scar after liquid crystal lubrication testing.

The surface of the PI film before friction was imaged using a white light interferometer, as shown in Figure 6a. The PI surface was relatively smooth and uniform, with no obvious texture, indicating that it was not affected by mechanical action. Figure 6b shows the surface morphology of the PI film after dry friction. Figure 6c shows the surface morphology of the PI film after liquid crystal lubrication testing under the same conditions as in Load of 25 N Section. The surface of the PI film showed obvious grooves aligned with the friction direction, which may be related to the orientation of the molecular chains. Further analysis can be conducted using polarized light microscopy. This indicates that friction can effectively change the surface morphology of the PI film and may also alter the arrangement of the PI molecules on the surface.

#### 3.3.2. Polarized Light Microscopy

**Figure 7:**
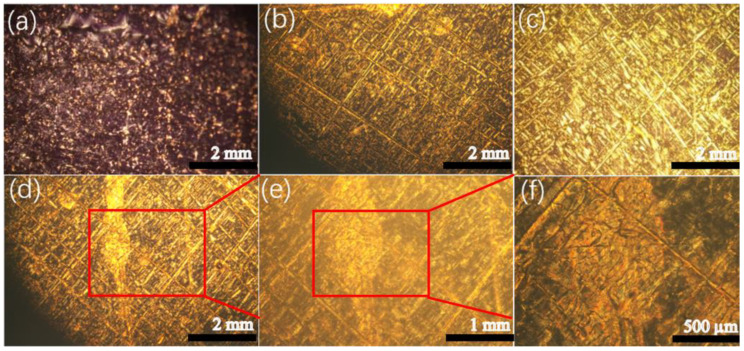
Polarized light microscopy images of the PI film. (**a**) Unfrictioned area of the original PI (5×); (**b**) back of the PI film (5×); (**c**) wear scar after dry friction under a 5 N load (5×); (**d**) wear scar after liquid crystal lubrication testing under a 90 N load (5×); (**e**) wear scar after liquid crystal lubrication testing under a 90 N load (10×); (**f**) wear scar after liquid crystal lubrication testing under a 90 N load (20×).

The morphology of the PI film was observed using polarized light microscopy. Before testing, the PI film was peeled off from the PI&PEEK sample and cut into an appropriate size for placement on a glass slide. The surface morphology of the unfrictioned PI film observed under a polarized light microscope is shown in Figure 7a. It appears relatively smooth, with an overall dark field and a few yellow bright spots. This is because polyimide is an amorphous material and does not exhibit optical anisotropy. Additionally, the surface of the unfrictioned film is quite smooth, resulting in minimal light scattering, which further reduces the brightness under the polarized light microscope. Therefore, it typically appears as a dark field under crossed polarizers. The few yellow bright spots are caused by external friction during the process of peeling the PI film from the PEEK substrate. The friction induces the orientation of some PI molecules along the friction direction, making them anisotropic and enhancing the birefringence phenomenon, thus producing a few yellow bright spots. Before synthesizing the PI film on the PEEK substrate, the PEEK surface was roughened with 180-grit sandpaper to enhance the adhesion between the PI film and the PEEK substrate. However, the rough surface also caused the back of the PI film to be rough, as shown in Figure 7b, where the back of the unfrictioned PI film showed cross-hatched scratches. In Figure 7c, the phenomenon of the PI film after dry friction under a 5 N load is shown. Compared to the unfrictioned areas, the friction traces on the PI film appear brighter. This is because friction increases the surface roughness of the friction traces, enhancing light scattering. At the same time, the friction causes the PI to orient along the friction direction, strengthening the birefringence phenomenon and making the friction traces appear brighter. The red box in Figure 7d–f show the wear scar on the PI film after liquid crystal lubrication testing under a load of 90 N and a speed of 200 rpm at different magnifications. The anisotropic LCs adhered to the grooves of the wear scar on the PI film. In summary, friction caused the PI film to orient along the friction direction, forming anisotropic PI, and the LCs adhered to the wear scars on the PI film and oriented accordingly.

#### 3.3.3. Raman Spectroscopy

**Figure 8:**
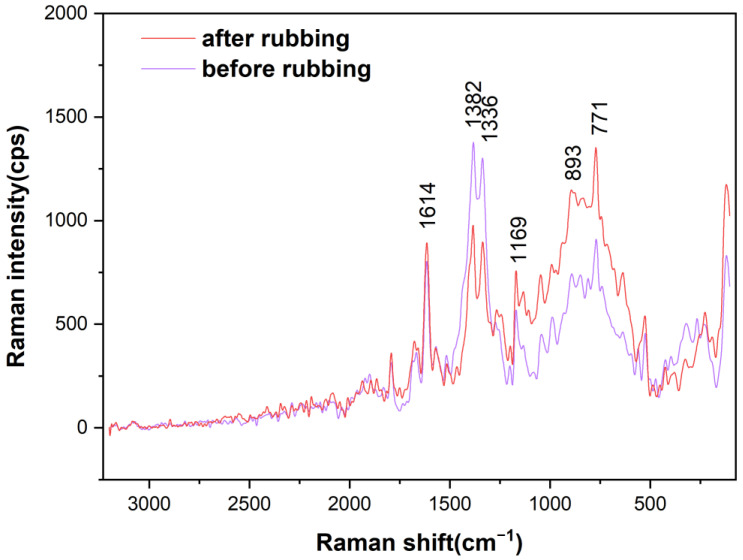
Raman spectra of the PI film before and after friction.

Figure 8 shows the Raman spectra of the PI film before and after liquid crystal lubrication testing under the same conditions as in Load of 25 N Section. The peak positions did not shift after friction, indicating that the molecular structure of the PI film was not significantly affected by friction. However, the intensity of the aromatic ring characteristic peaks (C=C stretching vibration at 1614 cm^−1^ and C-H bending vibration at 1169 cm^−1^) increased after friction, while the intensity of the imide characteristic peaks (C-N stretching vibration at 1382 cm^−1^ and C-H bending vibration at 1336 cm^−1^) decreased. This may be due to the adsorption of lubricant molecules on the wear scar of the PI film, altering the interfacial structure of the friction system.

## 4. Conclusions

Coating PEEK with a PI film significantly enhances its tribological performance by reducing COF and improving the stability and service life of dynamic seals. Compared to the GCr15/PEEK friction pair, the GCr15/PI&PEEK pair with a liquid crystal lubricant achieved a superlubricity-level COF (<0.01), representing an 83.3% reduction. This improvement is attributed to the PI surface facilitating the alignment of 5CB lubricant molecules along the friction direction, forming a stable, low-viscosity lubricating film. The system’s COF first increases and then decreases with increasing load but rises again once the load exceeds the stable load-bearing limit of the lubricating film (65–95 N). Excessive loads (>25 N) can diminish the influence of speed on the stability of the friction system.

The friction system can be further optimized by enhancing PI&PEEK interfacial adhesion through plasma treatment, interfacial agents, or nanofillers. Incorporating nanofibers like carbon nanotubes or graphene can also strengthen the PI layer, improving wear resistance and adaptability under harsh conditions. Additionally, using advanced lubricants such as ionic or polymer ionic liquids may further improve tribological performance.

## Data Availability

The original contributions presented in this study are included in the article. Further inquiries can be directed to the corresponding authors.

## References

[1] Perry S.S., Tysoe W.T. (2005). Frontiers of fundamental tribological research. Tribol. Lett..

[2] Holmberg K., Erdemir A. (2017). Influence of tribology on global energy consumption, costs and emissions. Friction.

[3] Woydt M. (2021). The importance of tribology for reducing CO_2_ emissions and for sustainability. Wear.

[4] Zheng Z., Guo Z., Liu W., Luo J. (2023). Low friction of superslippery and superlubricity: A review. Friction.

[5] Hirano M., Shinjo K. (1990). Atomistic locking and friction. Phys. Rev. B.

[6] Hirano M., Shinjo K., Kaneko R., Murata Y. (1991). Anisotropy of frictional forces in muscovite mica. Phys. Rev. Lett..

[7] Martin J.M., Donnet C., Le Mogne T., Epicier T. (1993). Superlubricity of molybdenum disulphide. Phys. Rev. B.

[8] Li Q., Newberg J.T., Walter E.C., Hemminger J.C., Penner R.M. (2004). Polycrystalline Molybdenum Disulfide (2H−MoS2) Nano- and Microribbons by Electrochemical/Chemical Synthesis. Nano Lett..

[9] Sano N., Wang H., Chhowalla M., Alexandrou I., Amaratunga G.A.J., Naito M., Kanki T. (2003). Fabrication of inorganic molybdenum disulfide fullerenes by arc in water. Chem. Phys. Lett..

[10] Jiménez I., Jankowski A.F., Terminello L.J., Sutherland D.G.J., Carlisle J.A., Doll G.L., Tong W.M., Shuh D.K., Himpsel F.J. (1997). Core-level photoabsorption study of defects and metastable bonding configurations in boron nitride. Phys. Rev. B.

[11] Cumings J., Zettl A. (2000). Low-Friction Nanoscale Linear Bearing Realized from Multiwall Carbon Nanotubes. Science.

[12] Hirano M., Shinjo K., Kaneko R., Murata Y. (1997). Observation of Superlubricity by Scanning Tunneling Microscopy. Phys. Rev. Lett..

[13] Dienwiebel M., Verhoeven G.S., Pradeep N., Frenken J.W.M., Heimberg J.A., Zandbergen H.W. (2004). Superlubricity of Graphite. Phys. Rev. Lett..

[14] Hod O., Meyer E., Zheng Q., Urbakh M. (2018). Structural superlubricity and ultralow friction across the length scales. Nature.

[15] Song Y., Mandelli D., Hod O., Urbakh M., Ma M., Zheng Q. (2018). Robust microscale superlubricity in graphite/hexagonal boron nitride layered heterojunctions. Nat. Mater..

[16] Qu C., Wang K., Wang J., Gongyang Y., Carpick R.W., Urbakh M., Zheng Q. (2020). Origin of Friction in Superlubric Graphite Contacts. Phys. Rev. Lett..

[17] Song Y., Qu C., Ma M., Zheng Q. (2020). Structural Superlubricity Based on Crystalline Materials. Small.

[18] Huang X., Li T., Wang J., Xia K., Tan Z., Peng D., Xiang X., Liu B., Ma M., Zheng Q. (2023). Robust microscale structural superlubricity between graphite and nanostructured surface. Nat. Commun..

[19] Huang X., Xiang X., Li C., Nie J., Shao Y., Xu Z., Zheng Q. (2025). Electrostatic in-plane structural superlubric actuator. Nat. Commun..

[20] Ge X., Li J., Zhang C., Wang Z., Luo J. (2018). Superlubricity of 1-Ethyl-3-methylimidazolium trifluoromethanesulfonate Ionic Liquid Induced by Tribochemical Reactions. Langmuir.

[21] Lebedeva O., Kultin D., Kustov L. (2024). Polymeric ionic liquids: Here, there and everywhere. Eur. Polym. J..

[22] Liu M., Ni J., Zhang C., Wang R., Cheng Q., Liang W., Liu Z. (2024). The Application of Ionic Liquids in the Lubrication Field: Their Design, Mechanisms, and Behaviors. Lubricants.

[23] Li J., Zhang C., Luo J. (2011). Superlubricity Behavior with Phosphoric Acid–Water Network Induced by Rubbing. Langmuir.

[24] Chen Z., Liu Y., Zhang S., Luo J. (2013). Controllable Superlubricity of Glycerol Solution via Environment Humidity. Langmuir.

[25] Ge X., Li J., Luo R., Zhang C., Luo J. (2018). Macroscale Superlubricity Enabled by the Synergy Effect of Graphene-Oxide Nanoflakes and Ethanediol. ACS Appl. Mater. Interfaces.

[26] Wu S., He F., Xie G., Bian Z., Luo J., Wen S. (2018). Black Phosphorus: Degradation Favors Lubrication. Nano Lett..

[27] Wang H., Liu Y., Liu W., Liu Y., Wang K., Li J., Ma T., Eryilmaz O.L., Shi Y., Erdemir A. (2019). Superlubricity of Polyalkylene Glycol Aqueous Solutions Enabled by Ultrathin Layered Double Hydroxide Nanosheets. ACS Appl. Mater. Interfaces.

[28] Liu Y., Li J., Li J., Yi S., Ge X., Zhang X., Luo J. (2021). Shear-Induced Interfacial Structural Conversion Triggers Macroscale Superlubricity: From Black Phosphorus Nanoflakes to Phosphorus Oxide. ACS Appl. Mater. Interfaces.

[29] Zhang S., Liu Y., Luo J. (2014). In situ observation of the molecular ordering in the lubricating point contact area. J. Appl. Phys..

[30] Gao M., Ma L., Luo J. (2016). Effect of Alkyl Chain Length on the Orientational Behavior of Liquid Crystals Nano-Film. Tribol. Lett..

[31] Li J., Zhang C., Luo J. (2013). Superlubricity Achieved with Mixtures of Polyhydroxy Alcohols and Acids. Langmuir.

[32] Ge X., Li J., Zhang C., Luo J. (2018). Liquid Superlubricity of Polyethylene Glycol Aqueous Solution Achieved with Boric Acid Additive. Langmuir.

[33] Gao X., Chen H., Lv S., Zhang Z., Wang T. (2021). Preliminary Study of the Superlubricity Behavior of Polyimide-Induced Liquid Crystal Alignment. J. Tribol..

[34] Gao X., Cheng Y., Shi M., Chen H., Wu L., Wang T. (2023). Design of Superlubricity System Using Si_3_N_4_/Polyimide as the Friction Pair and Nematic Liquid Crystals as the Lubricant. Polymers.

[35] Gao X., Chen H., Yan H., Huang C., Wu L., Wang T. (2023). Superlubricity by polyimide-induced alignment. Friction.

[36] Bi H.-S., Zhi X.-X., Wu P.-H., Zhang Y., Wu L., Tan Y.-Y., Jia Y.-J., Liu J.-G., Zhang X.-M. (2020). Preparation and Characterization of Semi-Alicyclic Polyimide Resins and the Derived Alignment Layers for Liquid Crystal Display Technology. Polymers.

[37] Son S.-R., An J., Choi J.-W., Lee J.H. (2021). Fabrication of TiO2-Embedded Polyimide Layer with High Transmittance and Improved Reliability for Liquid Crystal Displays. Polymers.

[38] Tatsumi G., Ratoi M., Shitara Y., Hasegawa S., Sakamoto K., Mellor B.G. (2021). Mechanism of oil-lubrication of PEEK and its composites with steel counterparts. Wear.

[39] Lv X., Yang S., Pei X., Zhang Y., Wang Q., Wang T. (2024). An investigation of the microstructure and tribological behavior of polyether ether ketone composites fabricated by extrusion-based additive manufacturing. Polym. Int..

[40] Li Y., Wang Z., Cui X., Han X., Zhang J. (2024). Tribological Properties of PEEK and Its Composite Material under Oil Lubrication. Lubricants.

[41] Padhan M., Marathe U., Bijwe J. (2020). Tribology of Poly(etherketone) composites based on nano-particles of solid lubricants. Compos. Part B Eng..

[42] Guru S.R., Panda S., Kumar P., Sarangi M. (2024). A study on tribological performances of PEEK and PTFE based composites with MoS reinforcements. Polym. Compos..

[43] Ma H., Han H., Zhao X., Ma J., Qu X., Lou X., Suonan A., Lei B., Zhang Y. (2023). Engineering Multifunctional Polyether Ether Ketone Implant: Mechanics-Adaptability, Biominerialization, Immunoregulation, Anti-Infection, Osteointegration, and Osteogenesis. Adv. Healthc. Mater..

[44] Zhao X., Xiong D., Liu Y. (2018). Improving surface wettability and lubrication of polyetheretherketone (PEEK) by combining with polyvinyl alcohol (PVA) hydrogel. J. Mech. Behav. Biomed. Mater..

[45] (2022). Standard Test Method for Peel Adhesion of Pressure-Sensitive Tape.

